# Contrast-Enhanced Cone-Beam Breast CT: An Analysis of Diagnostic Value in Predicting Breast Lesion With Rim Enhancement Malignancy

**DOI:** 10.3389/fonc.2022.868975

**Published:** 2022-05-24

**Authors:** Xin Zhao, Jun Yang, Yang Zuo, Wei Kang, Hai Liao, Zhong-Tao Zheng, Dan-Ke Su

**Affiliations:** Department of Radiology, Guangxi Medical University Cancer Hospital, Nanning, China

**Keywords:** contrast-enhanced cone-beam breast computed tomography, clustered rim enhancement, qualitative morphological enhancement parameters, quantitative enhancement parameters, diagnostic accuracy

## Abstract

**Background:**

The objective of the current study was to investigate the diagnostic value of contrast-enhanced cone-beam breast computed tomography (CE-CBBCT) for breast lesion with rim enhancement (RE).

**Methods:**

All 36 patients were examined by non-contrast (NC-CBBCT) and contrast-enhanced CBBCT (CE-CBBCT) after contrast media (CM) injection. Qualitative morphological enhancement parameters and quantitative enhancement parameters were compared between malignant and benign groups. Multivariable logistic regression analysis was performed to identify independent factors that could predict breast lesion with RE malignancy. Receiver operating curve (ROC) was used to evaluate prediction performance.

**Results:**

A total of 36 patients with 40 lesions underwent breast CE-CBBCT were enrolled. There were significant differences in most qualitative morphological enhancement parameters between the two groups. A multivariate logistic regression model showed that △standardized HU (INR_phase 2_−INR_preCM_) [odds ratio (OR) = 1.148, 95% CI = 1.034–1.276, *p* = 0.01] and △standardized HU (RP_phase 2_ − RP_phase 1_) (OR = 0.891, 95% CI = 0.814–0.976, *p* = 0.013) were independent indicators in predicting breast lesion with RE malignancy. △standardized HU (INR_phase 2_ − INR_preCM_) combined with △standardized HU (RP_phase 2_ − RP_phase 1_) showed significant larger area under the receiver operating curve (AUC) and higher sensitivity than each alone (*p* < 0.001, AUC = 0.932, sensitivity = 92.59%, specificity = 92.31%). The regression equation of the prediction model was as follows: Logit (*p*) = 0.351 + 0.138X × △standardized HU (INR_phase 2_ − INR_preCM_) − 0.115 × △standardized HU (RP_phase 2_ − RP_phase 1_).

**Conclusion:**

With the observation of qualitative morphological enhancement parameters and the comparison of quantitative enhancement parameters of CBBCT, a reliable basis for the diagnostic accuracy in predicting breast lesion with RE could be provided. These conclusions should be verified in large, well-designed studies.

## Introduction

Breast lesion with rim enhancement (RE) is a special type of lesion with more enhancement in the edge than in the central region of the lesion through dynamic enhanced magnetic resonance imaging (MRI) scanning. Previous studies (1, 2) have indicated that REs are highly suggestive of malignant lesion. However, in clinical practice, REs may also appear in some of those benign lesions and often partially overlap with the malignant lesions, thus affecting the accuracy of qualitative diagnosis of lesions. With the rapid development of imaging technology, cone-beam breast computed tomography (CBBCT), as a new dedicated breast CT imaging technology based on cone-beam X-ray and flat panel detector (3, 4), has opened a new chapter in breast imaging diagnosis. In particular, the diagnostic effectiveness of contrast-enhanced cone-beam breast computed tomography (CE-CBBCT) is close to MRI (5), providing a new direction for the diagnosis of breast diseases. In the current study, the diagnostic value of CE-CBBCT in predicting breast lesion with RE malignancy was investigated.

## Materials and Methods

### Ethics Statement

This study was conducted in accordance with the Declaration of Helsinki and approved by the Institutional Review Board of Guangxi Medical University Cancer Hospital. Written informed consent was given by all participants for their clinical records to be used in this study.

### Patients

Patients who received CE-CBBCT as standard of care from July 2019 to October 2019 were retrospectively reviewed

The inclusion criteria were as follows: (1) the affected breast was imaged before breast biopsy, lumpectomy, and chemoradiotherapy; (2) CE-CBBCT scan was performed, and mass lesions with RE was detected by radiologist in the affected breast; and (3) the malignancy of the mass lesion was proven by pathology after biopsy or surgery.

A total of 36 patients with 40 lesions underwent breast CE-CBBCT were enrolled.

### CBBCT Scanning

CBBCT examinations were performed using a dedicated flat-panel breast CT system (Koning Breast CT, CBCT 1000, Koning Corporation). The CBBCT system has been approved by the Food and Drug Administration (FDA) and the National Food and Drug Administration of China for diagnostic breast imaging. The CBBCT examinations were performed with a constant tube voltage of 49 kVp and variable tube currents (between 50 and 160 mA) depending on breast size and density (4, 6). Tube current was automatically selected after an initial scout image acquisition and kept constant for pre- and post-contrast CBBCT imaging. The patients took prone position with their arms raising, keeping the breasts naturally pendant at the center of the imaging field. The position was not changed during the whole examination. After the initial pre-contrast CBBCT scanning, ioversol contrast media (CM) (320 mgI/ml) was injected intravenously with a dual-chamber power injector at the flowrate of 2 ml/s and at the dose of 1.5–2.0 ml/kg. Two separate post-contrast CBBCT scans were performed at 60 s (phase 1) and 110 s (phase 2) after the injection of CM.

### Image and Data Analyses

Image analyses were performed by two breast radiologists who were highly experienced in breast imaging including CBBCT. Both radiologists were blinded to clinicopathological and other imaging modality findings. When the diagnosis was inconsistent, the final decision was based on the agreement of the two radiologists. Koning Breast CT Image Viewer workstation was used to observe the qualitative morphological enhancement parameters and measure the quantitative enhancement parameters. CBBCT intensity was measured in Hounsfield units (HUs). Qualitative morphological enhancement parameters were described based on MRI breast imaging reporting and data system (BI-RADS), including the overall shape of the rim enhancement, the situation of rim paries (outer margin of the rim paries, border of outer margin, inner margin of the rim paries, border of inner margin, and uniformity of the rim paries), and peripheral vascular signs. Quantitative enhancement parameters included were as follows. First is the maximum thickness difference of the rim paries: the coronal plane of the lesion in the phase 1 of CE-CBBCT was selected to measure the thickest and thinnest diameter of rim paries; then, the difference between the two was calculated. Second is the △standardized CT value (HU): regions of interest (ROI) were selected at the same positions in different phases (non-enhanced and two-phase enhanced scans) of the rim paries, the inner of the rim, and the fat for CT value measurement (ROI area was 2–5 mm^2^). When selecting an ROI for CT value measurement, it should be noted that (1) the ROI of the rim paries is selected in the area where the rim paries was significantly enhanced in the enhanced scan image, and the ROI in the non-enhanced scan image should correspond with it; (2) the ROI of the inner of the rim is selected in the area where the inner of the rim was not enhanced or not obviously enhanced in the enhanced scan image, and the ROI in the non-enhanced scan image should correspond with it; (3) when selecting the ROI of the fat, glands, blood vessels, skin, and other structures should be avoided; and (4) the measurement is performed on the image of coronal section with a thickness of 0.27 mm. After measuring the CT value, referring to the calculation methods of the enhancement parameters of Liu et al. (7) and Uhlig et al. (8), △standardized HUs were calculated according to the following formula:


$$\text{Formula }1:\Delta {\text{standardized HU (INR}}_{\text{phase }1}-{\text{ INR}}_{\text{preCM}}\text{) }={\text{ HU(INR}}_{\text{phase }1}-{\text{ INR}}_{\text{preCM}}\text{) }-{\text{ HU(fat}}_{\text{phase }1}-{\text{ fat}}_{\text{preCM}}).$$

$$\text{Formula }2:\Delta {\text{standardized HU (INR}}_{\text{phase }2}-{\text{ INR}}_{\text{preCM}}\text{) }={\text{ HU(INR}}_{\text{phase }2}-{\text{ INR}}_{\text{preCM}}\text{) }-{\text{ HU(fat}}_{\text{phase }2}-{\text{ fat}}_{\text{preCM}}).$$

$$\text{Formula }3:\Delta \text{standardized HU }({\text{INR}}_{\text{phase }2}-{\text{ INR}}_{\text{phase }1)}={\text{ HU(INR}}_{\text{phase }2}-{\text{ INR}}_{\text{phase }1}\text{) }-{\text{ HU(fat}}_{\text{phase }2}-{\text{ fat}}_{\text{phase }1}).$$

$$\text{Formula }4:\Delta {\text{standardized HU (RP}}_{\text{phase }1}-{\text{ RP}}_{\text{preCM}}\text{) }={\text{ HU(RP}}_{\text{phase }1}-{\text{ RP}}_{\text{preCM}}\text{) }-{\text{ HU(fat}}_{\text{phase }1}-{\text{ fat}}_{\text{preCM}}).$$

$$\text{Formula }5:\Delta {\text{standardized HU (RP}}_{\text{phase }2}-{\text{ RP}}_{\text{preCM}}\text{) }={\text{ HU(RP}}_{\text{phase }2}-{\text{ RP}}_{\text{preCM}}\text{) }-{\text{ HU(fat}}_{\text{phase }2}-{\text{ fat}}_{\text{preCM}}).$$

$$\text{Formula }6:\Delta {\text{standardized HU (RP}}_{\text{phase }2}-{\text{ RP}}_{\text{phase }1}\text{) }={\text{ HU(RP}}_{\text{phase }2}-{\text{ RP}}_{\text{phase }1}\text{) }-{\text{ HU(fat}}_{\text{phase }2}-{\text{ fat}}_{\text{phase }1}).$$

### Statistical Analysis

SPSS Version 25.0 (IBM, Armonk, NY, USA) was used. Continuous variables are presented as mean ± standard deviation (SD) as measure of dispersion. The normality assumption of continuous variables was tested *via* the Shapiro–Wilks test. Continuous variables that did not conform to normal distribution are expressed as quartiles, which were presented as median (P25, P75). Categorical variables are presented as absolute number and percent.

Qualitative morphological enhancement parameters between malignant and benign groups were compared by using χ^2^ or Fisher’s exact tests. Quantitative enhancement parameters between malignant and benign groups were compared by Student’s t-test or Mann–Whitney U-tests. Those quantitative enhancement parameters with *p* < 0.1 in the univariate analysis were included in the multivariable logistic regression analysis using forward:LR to identify independent factors that could predict breast lesion with RE malignancy. Diagnostic accuracy was assessed lesion based *via* test sensitivity, specificity, and area under the receiver operating curve (AUC) separately for those quantitative enhancement parameters with *p* < 0.05 in the multivariable logistic regression analysis by calculating the receiver operating curve (ROC). A *p* value < 0.05 was considered significant.

## Results

### Patient Characteristics

The clinicopathological data of the patients included are presented in **Table 1**. A total of 36 patients with 40 lesions fulfilled the inclusion criteria. NC-CBBCT, post-CM CE-CBBCT scans at 60 s (phase 1), and 110 s (phase 2) were performed in all patients. All patients were female. The age of the patients ranged from 35 to 64 years, and the median age was 46 years. A total of 13 benign lesions (6 were proliferative lesions with inflammation, 3 were purulent inflammation, 3 were plasma cell mastitis, and 1 was fibroadenoma) were found in 11 patients. A total of 27 malignant lesions (all of them were invasive ductal carcinoma: 3 were luminal A subtype, 6 were luminal B subtype, 11 were Her-2-positive subtype, and 7 were triple negative subtype) were found in 25 patients.

**Table 1:** The clinicopathological data and molecular pathological subtypes of these patients.

	Malignant group (n = 25)	Benign group (n = 11)	*p*-value
Number of lesions (n)	27	13	
Age (years)	48.36 ± 6.775	43.36 ± 4.433	0.032
Menstrual status (n)			0.387
Non-menopause	18	10	
Menopause	7	1	
Gland type (n)			0.411
Non-compact	12	4	
compact	13	7	
Size of lesion (cm)	2.24 ± 0.93	1.60 ± 0.58	0.005

Data are shown as the mean ± SD or number of patients.

### Radiation Dose

The radiation doses of the 36 patients enrolled in this study ranged from 15.3 to 22.7 mGy, with a mean dose of 17.73 ± 1.53 mGy.

### Comparison of the Qualitative Morphological Enhancement Parameters Between Malignant and Benign Groups

**Figure 1:**
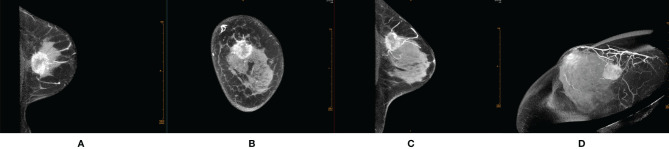
Female, 48 years old, invasive ductal carcinoma in the left breast. **(A)** Transverse section. **(B)** Coronal section. **(C)** Median sagittal section. **(D)** MIP reconstruction images in phase 1 by CE-CBBCT. Those images showed an irregular rim-shaped enhanced mass with uneven thickness of rim paries, spicular outer margin of the rim paries, unsmooth and undefinite inner margin of the rim paries, and positive peripheral vascular signs (increased, thickened blood vessels around the mass and partially connected to it) in the upper quadrant of the left breast at about 12 o’clock. CE-CBBCT, contrast-enhanced CBBCT (CE-CBBCT); MIP, maximum intensity projection.

**Figure 2:**
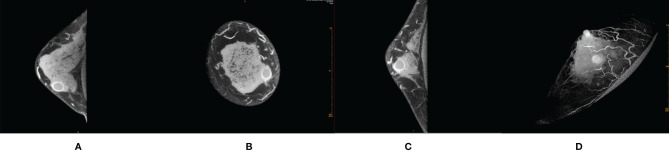
Female, 32 years old, cystic fibrosis with inflammation in right breast. **(A)** Transverse section. **(B)** Coronal section. **(C)** Median sagittal section. **(D)** MIP reconstruction images in phase 1 by CE-CBBCT. Those image showed a circular rim-shaped enhanced mass with uniform thickness of rim paries, smooth and definite inner and outer margin of the rim paries, and negative peripheral vascular signs (without obvious increased and thickened blood vessels around the mass) in the inner and lower quadrant of the right breast at about 4 o’clock. CE-CBBCT, contrast-enhanced CBBCT (CE-CBBCT); MIP, maximum intensity projection.

Most of the malignant lesions with REs showed irregular shape, irregular/spicula outer margin of the rim paries, unsmooth and indefinite inner margin of the rim paries, uneven thickness of the rim paries, and positive peripheral vascular sign (**Figure 1**). Most of the benign lesions showed round/quasi-circular shape, smooth/lobulate outer margin of the rim paries, smooth and definite inner margin of the rim paries, and uniform thickness of the rim paries (**Figure 2**). There were significant differences in most signs between the two groups except for one (border of outer margin) (**Table 2**).

**Table 2:** Morphological enhancement signs of the breast lesions with rim enhancements by CE-CBBCT.

Morphological enhancement signs	Malignant group (n = 27)	Benign group (n = 13)	χ^2^	*p-*value
Shape			9.548	0.002
Round/quasi-circular shape	11	12		
Irregular	16	1		
Outer margin of the rim paries			15.506	<0.001
Smooth/lobulate	7	12		
Irregular/spicular	20	1		
Border of outer margin			0.105	0.746
Clear	16	7		
Unclear	11	6		
Inner margin of the rim paries			13.713	<0.001
Smooth and definite	2	8		
Unsmooth and undefinite	25	5		
Border of inner margin			19.551	<0.001
Clear	5	12		
Unclear	22	1		
Uniformity of the rim paries			14.661	<0.001
Uniform thickness	0	6		
Uneven thickness	27	7		
Peripheral vascular signs			4.569	0.033
Positive	18	4		
Negative	9	9		

CE-CBBCT, contrast-enhanced cone-beam breast computed tomography.

### Univariate Analysis and Multivariable Logistic Regression Analysis of the Quantitative Enhancement Parameters

Those quantitative enhancement parameters including thickness difference of the rim paries, △standardized HU (INR_phase 1_ − INR_preCM_), △standardized HU (INR_phase 2_ − INR_preCM_), △standardized HU (RP_phase 1_ − RP_preCM_), and △ standardized HU (RP_phase 2_ − RP_phase 1_) in malignant group were significantly higher than that in the benign group (**Table 3**).

**Table 3:** Comparison of the quantitative enhancement parameters between malignant and benign groups by CE-CBBCT.

Parameters	Malignant group (n = 27)	Benign group (n = 13)	t or U	*p-*value
Maximum thickness difference of the rim paries (cm)	0.35 (0.2–0.7)	0.06 (0.04–0.07)	U = 37.500	<0.001
△standardized HU (INR_phase 1_ − INR_preCM_) (Hu)	13.56 ± 17.26	0.37 ± 12.53	t = −2.455	0.019
△standardized HU (INR_phase 2_ − INR_preCM_) (Hu)	23.24 ± 28.26	3.81 ± 14.52	t = −2.325	0.026
△standardized HU (INR_phase 2_ − INR_phase 1)_ (Hu)	9.68 ± 22.05	3.44 ± 11.31	t = −0.957	0.345
△standardized HU (RP_phase 1_ − RP_preCM_) (Hu)	85.9 ± 42.18	55.52 ± 34.14	t = 0.260	0.03
△standardized HU (RP_phase 2_ − RP_preCM_) (Hu)	84.62 ± 41.43	75.08 ± 37	t = −0.705	0.485
△standardized HU (RP_phase 2_ − RP_phase 1_) (Hu)	−1.27 ± 33.89	19.56 ± 16.22	t = 2.094	0.043

CE-CBBCT, contrast-enhanced cone-beam breast computed tomography; HU, ounsfifield Units; INR, the inner of the rim; RP, rim paries.

The quantitative enhancement parameters with *p* < 0.1 in univariate analysis above were included in the multivariable logistic regression analysis to identify independent factors that could predict breast lesion with RE malignancy. Results showed that △standardized HU (INR_phase 2_ − INR_preCM_) (OR = 1.148, 95% CI =1.034–1.276, *p* = 0.01) and △standardized HU (RP_phase 2_ − RP_phase 1_) (OR = 0.891, 95% CI =0.814–0.976, *p* = 0.013) were independent indicators in predicting breast lesion with RE malignancy (**Table 4**).

**Table 4:** Multivariable logistic regression analysis to identify independent factors that could predict breast lesion with RE malignancy.

Parameters	Coefficient	Standard error	Wald value	*p*-value	OR (95%CI)
△standardized HU (INR_phase 2_ − INR_preCM_) (Hu)	0.138	0.054	6.637	0.01	1.148 (1.034–1.276)
△ standardized HU (RP_phase 2_ − RP_phase 1_) (Hu)	−0.115	0.047	6.107	0.013	0.891 (0.814–0.976)

RE, rim enhancement; HU, Hounsfield units; INR, the inner of the rim; RP, rim paries; CI, confidence interval; OR, odds ratios.

### Diagnostic Accuracy of the Enhancement Parameters

Both △standardized HU (INR_phase 2_ − INR_preCM_) and △standardized HU (RP_phase 2_ − RP_phase 1_) alone showed comparable AUC, sensitivity, and specificity for assessment of breast lesions malignancy. While △standardized HU (INR_phase 2_ − INR_preCM_) combined with △standardized HU (RP_phase 2_ − RP_phase 1_) showed significant larger AUC and higher sensitivity than each alone. The regression equation of the prediction model for combined both was as follows: Logit (*p*) = 0.351 + 0.138X × △standardized HU (INR_phase 2_ − INR_preCM_) − 0.115 × △standardized HU (RP_phase 2_ − RP_phase 1_) (**Table 5**).

**Table 5:** Summary of diagnostic accuracy of different parameters for predicting breast lesion with RE malignancy.

Modality	AUC	*p*-value	Cutoff	Youden’s index	Sensitivity	Specificity
△standardized HU (INR_phase 2_ − INR_preCM_) (Hu)	0.732 (0.569–0.860)	0.026	≤−3.3	0.519	51.85%	100%
△standardized HU (RP_phase 2_ − RP_phase 1_) (Hu)	0.719 (0.555–0.850)	0.019	>14.4	0.405	48.15%	92.31%
△standardized HU (INR_phase 2_ − INR_preCM_) combined with △standardized HU (RP_phase 2_ − RP_phase 1_) (Hu)	0.932 (0.805–0.987)	<0.001	>0.537	0.849	92.59%	92.31%

RE, rim enhancement; HU, Hounsfifield Units; INR, the inner of the rim; RP, rim paries; AUC, area under the receiver-operating curve.

## Discussion

REs may be found in both benign and malignant lesions. Most previous studies mainly focused on the differential diagnosis of RE in MRI technology, and most researchers believed that the post-CM morphological characteristics of lesions after scanning are vital basis for qualitative diagnosis (9–12). As a new type of equipment dedicated to breast imaging, CBBCT has the advantages including fast scanning speed and high image quality. It can obtain three-dimensional images with high spatial resolution and contrast from all directions and multiple perspectives without displacement and deformation. In addition, it has strong ability to display lesions of microcalcifications and soft tissue and to improve the detection of post-CM lesions and highlight their morphological characteristics after scanning (3, 5).

In the current study, the morphological enhancement parameters of breast lesions with REs by CE-CBBCT were analyzed, and the results indicated that there were significant differences in overall shape of the REs and the situation of rim paries (outer margin of the rim paries, border of outer margin, inner margin of the rim paries, border of inner margin, and uniformity of the rim paries, and peripheral vascular signs) between malignant and benign lesions. These findings were consistent with the conclusions of most previous studies on the morphological characteristics of breast lesions with REs by MRI scanning (9–15). The difference in post-CM morphology between benign and malignant lesions with REs is mainly related to the difference in their biological behavior and pathological basis (16). The rim paries of benign lesions of which the growth rates are slow and uniform mostly consist of abscess wall, cyst wall with dilated duct wall, or inflammatory cell infiltration. Therefore, the corresponding imaging characteristics of the REs in benign lesions are mostly round/quasi-circular shape with smooth/lobulate outer margin and definite inner margin, and uniform thickness of their rim paries. The rim paries of malignant lesions of which the growth rates are fast and nonuniform consist of tumor cells that are high value added and heterogeneous. In addition, much vascular tumor angiogenesis around the tumor body are induced by endothelial growth factor (VEGF). Thus, the corresponding imaging characteristics of the REs in malignant lesions are mostly irregular shape with irregular/spicula outer margin, unsmooth and indefinite inner margin, uneven thickness of their rim paries, and positive peripheral vascular sign. The positive peripheral vascular sign is common. In this study, there was no significant difference between benign and malignant groups in the border of outer margin of the rim paries. The proportion of benign lesions with unclear border was equivalent to that with clear border of outer margin (6:7). A similar proportion was observed in malignant group (unclear border:clear border, 11:16). The proportion of benign lesions with blurred outer boundary was equivalent to that with clear outer boundary (6:7) to that of malignant group (11:16). This may be due to the fact that the benign group in the current study mainly consisted of inflammatory or benign lesions combined with inflammation, and the inflammatory edema or granulation tissue hyperplasia around the lesion caused by inflammation may be the reason for the unclear border of the outer margin of the rim paries between benign lesions and adjacent tissues.

In addition to the qualitative diagnosis of breast lesions by qualitative morphological enhancement parameters, the quantitative enhancement parameters of CE-CBBCT could reflect the hemodynamic characteristics of the lesions to a certain extent, so as to provide quantitative diagnostic basis for the identification of benign and malignant lesions. At present, there is no uniform standard for the time point setting of CE-CBBCT phases and the measurement methods of CE-CBBCT CT value in lesions all over the world (3, 5, 7, 8, 17). The traditional CT value is not suitable for cone-beam breast CT because the cone-line-beam imaging characteristics of cone-beam breast CT are different from those of conventional spiral CT. Our previous study (18) has shown that the stability of absolute CT value of CBBCT is lower than that of conventional spiral CT. Even in the same tissue of a breast, the corresponding absolute CT values of CBBCT in different positions of the breast differ. Therefore, in order to reduce the influence from the instability of absolute CT value, breast fat was used by researchers to standardize the CT value of CBBCT to get a △CT value in breast lesion (8). In this study, △CT value was used as the value of the density measurement for rim enhancement. Whether the relative CT value obtained by this method is more stable than the absolute CT value needs more experimental and theoretical confirmation in the future. Liu et al. (7) obtained the △CT value through calculation of the difference in CT value before and after injection of CM with single phase of post-CM scanning at 120 s. Uhlig et al. (8) obtained the △standardized CT value through calculation of the difference in CT value before and after injection of CM with two phases of post-CM scanning at 2 and 3 min, respectively. Referring to the calculation methods of the enhancement parameters of the two researchers above (7, 8), in the current study, with two phases of post-CM scanning at 60 and 110 s, respectively, a series of quantitative enhancement parameters by CE-CBBCT were obtained. In addition, the diagnostic value with different combinations of those quantitative enhancement parameters were compared to get the optimal diagnostic parameters. The results suggested that △standardized HU (INR_phase 2_ − INR_preCM_), as one of the parameters with differential diagnostic significance in multivariable logistic regression analysis, was higher in the malignant group than that in the benign group, indicating that the degree on enhancement of the inner rim in the malignant lesion was significantly higher than that in the benign lesion. These findings support the conclusion of some previous studies. In the study of Buadu et al. (19), REs were observed in nine cases of invasive cancer, of which seven cases were connective tissue in the central region but not necrotic components. The results of Liu et al. (20) showed that the microvascular density in the margin area of the malignant lesion was significantly higher than that in the central area, resulting in lower perfusion of CM in the central area than that in the margin area. This indicates that the appearance of REs in malignant tumors are related to the regional differences in distribution of tumor microvascular, resulting in delayed enhancement in the central region. The pathological basis of REs in benign lesions are mainly related to central liquefaction necrosis, mammary duct dilatation, or high degree of fibrous tissue hyperplasia, resulting in post-CM non-enhancement or low degree of enhancement in the central region (13). Our results also suggested that △standardized HU (INR_phase 2_ − INR_preCM_), as another one of the parameters with differential diagnostic significance in multivariable logistic regression analysis, is significantly lower in the malignant group than that in the benign group, which indicated that the degree of enhancement of RE in malignant lesion is lower in phase 2 than that in phase 1, and the degree of enhancement of RE in benign lesion is gradually enhanced from phase 1 to 2. The results are consistent with the fact that outflow type is often found in malignant lesions and gradual increase type is often found in malignant lesions by MRI enhancement curve of time signal. However, whether the significance of types in MRI enhancement curve of time signal for the qualitative diagnosis of breast lesion is applicable to CE-CBBCT has not been reported at present, highlighting the need for further verification.

In terms of radiation dose control, it is worth mentioning that CBBCT could precisely control X-ray-related technical parameters such as tube voltage, tube current, and output power, and could adjust the scanning protocol individually according to the type, size, characteristics of breast glands, and the clinical requirements. In addition, during the examination, the system’s self-shielding prevents the contralateral breast and other parts of the body from being exposed to radiation. Radiation dose of CBBCT reported in the literature varied (6, 21–24), with a minimum total radiation dose of 4 mGy and a maximum of 16.6 mGy. The variability in radiation doses may be related to X-ray technology-related factors, breast characteristics, and different scanning protocols (non-enhanced scan and non-enhanced scan combined with single- or multi-phase enhancement). At present, researchers have not reached consensus on scanning protocols in CE-CBBCT examinations. Regulatory agencies such as US Food and Drug Administration regulate the radiation dose of breast cancer screening (3 mGy per view) but does not set limit for breast cancer diagnostic workup. The standard-of-care procedure in the hospital takes one pre-contrast and two post-contrast scans to achieve preferable enhancement while keeping the radiation dose at a level safe for diagnostic patients. The total radiation dose of 36 patients enrolled ranged from 15.3 to 22.7 mGy, with a mean dose of 17.73 ± 1.53 mGy. According to the International Commission on Radiological Protection (ICRP) publication 103 (2007), the breast tissue effective dose weighting factor is 0.12. The effective dose level of this CE-CBBT exam is between 1.83 and 2.72 mSv, which is equivalent to 8–12 months of natural background radiation (25) and only 20% of a whole-body CT dose (26).

To the best of our knowledge, this is the first study investigating the diagnostic value of CE-CBBCT for breast lesion with RE using a combination of post-CM qualitative morphological enhancement parameters and quantitative enhancement parameters. The results were significant, and we hope they would provide a reference for future studies; nonetheless, the work has several limitations that may affect interpretation of the results. On the one hand, comparative study has not been performed between imaging findings and pathology. On the other hand, the sample size was relative small, and the few pathological types were covered. Therefore, in the future, the sample size should be further expanded, the pathological types should be increased, and the comparative study on pathological and imaging findings should be performed to improve the accuracy of the conclusions.

In conclusion, with the observation of qualitative morphological enhancement parameters and the comparison of quantitative enhancement parameters by CBBCT, a reliable basis for the diagnostic accuracy in predicting breast lesion with RE malignancy could be provided. However, these conclusions should be verified in large, well-designed studies.

## Data Availability Statement

The original contributions presented in the study are included in the article/supplementary material. Further inquiries can be directed to the corresponding author.

## Ethics Statement

This study was conducted in accordance with the Declaration of Helsinki and approved by the Institutional Review Board of Guangxi Medical University Cancer Hospital. The patients/participants provided their written informed consent to participate in this study. Written informed consent was obtained from the individual(s) for the publication of any potentially identifiable images or data included in this article.

## Author Contributions

Designed the study: XZ and D-KS. Collected participants: JY and YZ. Analyzed the data: WK and HL. Statistical analyses: XZ and Z-TZ. Wrote the manuscript: XZ. All authors contributed to the article and approved the submitted version.

## Conflict of Interest

The authors declare that the research was conducted in the absence of any commercial or financial relationships that could be construed as a potential conflict of interest.

## Publisher’s Note

All claims expressed in this article are solely those of the authors and do not necessarily represent those of their affiliated organizations, or those of the publisher, the editors and the reviewers. Any product that may be evaluated in this article, or claim that may be made by its manufacturer, is not guaranteed or endorsed by the publisher.

## Abbreviations

CE-CBBCT, contrast-enhanced cone-beam breast computed tomography; NC-CBBCT, non-contrast CBBCT; MRI, magnetic resonance imaging; HU, Hounsfield units; INR, the inner of the rim; RP, rim paries; CI, confidence interval; ORs, odds ratios; AUC, area under the receiver operating curve; ROC, receiver operating curve.
